# Course of DISease In patients reported to the Swedish CPAP Oxygen and VEntilator RegistrY (DISCOVERY) with population-based controls

**DOI:** 10.1136/bmjopen-2020-040396

**Published:** 2020-11-19

**Authors:** Andreas Palm, Krister Ågren, Ludger Grote, Mirjam Ljunggren, Bengt Midgren, Josefin Sundh, J Theorell-Haglöw, Magnus Ekström

**Affiliations:** 1 Department of Medical Sciences, Respiratory, Allergy and Sleep Research, Uppsala Universitet, Uppsala, Sweden; 2 Centre for Research and Development, Uppsala University/Region of Gävleborg, Gavle, Sweden; 3 Internal Medicine and Clinical Nutrition, Goteborgs Universitet, Goteborg, Sweden; 4 Department of Clinical Sciences, Division of Respiratory Medicine & Allergology, Lunds University Faculty of Medicine, Lund, Sweden; 5 Department of Respiratory Medicine, School of Medical Sciences, Örebro University, Örebro, Sweden; 6 Department of Clinical Sciences, Division of Respiratory Medicine & Allergology, Lund University, Lund, Sweden

**Keywords:** adult thoracic medicine, epidemiology, sleep medicine

## Abstract

**Purpose:**

Chronic hypoxic and hypercapnic respiratory failure and obstructive sleep apnoea (OSA) are chronic diseases associated with decreased quality of life and increased mortality. The rationale behind the set up the retrospective nationwide DISCOVERY cohort was to study several questions including disease course and risk factors for incident disease, impaired quality of life, hospitalisation risk and mortality in patients with chronic respiratory failure with long-term oxygen therapy (LTOT), long-term mechanical ventilation (LTMV) and obstructive sleep apnoea (OSA) on treatment with continuous positive airway pressure (CPAP).

**Participants and settings:**

Data from the national quality registry for respiratory insufficiency and sleep apnoea (Swedevox) and a population-based control group from Statistics Sweden were merged with governmental registries, the Swedish Cancer Registry, the Swedish Cause of Death Registry, the Swedish Drug registry, the Swedish National Patient Registry and the Swedish Dental Health Registry and with national quality registries for diabetes, rheumatic diseases (Swedish Rheumatology Quality Registry), stroke (RiksStroke), heart failure (RiksSvikt), acute heart infarction care (SwedeHeart) and intensive care (SIR) and with socioeconomic data from Statistics Sweden (SCB).

**Findings to date:**

The cohort comprises 25 804 unique patients with LTOT since 1987 (54.1% females, age 73.3±9.8 years, body mass index (BMI) 26.6±6.5 kg/m^2^), 8111 with LTMV since 1996 (48.6% women, age 60.6±16.9 years, BMI 32.9±10.8 kg/m^2^), 65 809 with OSA on CPAP since 2010 (29.5% women, age 57.2±12.5 years, BMI 31.9±6.2 kg/m^2^) and 145 224 persons in a population-based control group from same time span up to March 2018 (51.7% women, age 49.9±20.4 year, BMI 24.9±4.0 years).

**Future plans:**

In patients with chronic respiratory failure and sleep apnoea important questions regarding comorbidity burden, hospitalisation rate, mortality and treatment outcomes are still unexplored to a large extent. The DISCOVERY cohort will provide unique opportunities by its size and comprehensiveness to fill this clinically relevant gap of knowledge.

**Strengths and limitations of this study**

- This nationwide population-based cohort is unique internationally due to its size, its high nationwide coverage, high degree of data completeness and high validity of data.
- No patients are lost to follow-up due to cross-linkage with mandatory national registries.
- To assure a high level of completeness in the Swedevox registry, the number of requested variables to be reported into the Swedevox registry has been deliberately limited, and the data thus become less detailed.
- Difficulties adjusting for confounders is a limitation in all registry-based studies.
- This is counterbalanced, at least in part, by the size of the cohort and by cross-linkage with other registries.

## Introduction

Chronic hypoxic and hypercapnic respiratory failure and obstructive sleep apnoea (OSA) are associated with decreased quality of life and increased mortality.^1–7^ Chronic hypoxic failure is caused by pulmonary diseases such as chronic obstructive pulmonary disease (COPD) and idiopathic pulmonary fibrosis^8^ and can be treated with long-term oxygen therapy (LTOT).^9^ Chronic hypercapnic respiratory failure caused by hypoventilation is treated with long-term mechanical ventilation (LTMV).^10^ OSA is a highly prevalent disease of variable aetiology and continuous positive airway pressure (CPAP)^11^ is the first-line treatment in moderate-to-severe OSA. Large-scale outcome data are sparse in these patient groups with chronic diseases characterised by substantial respiratory distress. Despite high prevalence and substantial heathcare utilisation, several important questions on long-term treatment outcomes are not yet sufficiently studied.

In chronic hypoxic respiratory failure, two-thirds of the patients die of cardiovascular disease (CVD) and cancer.^12 13^ There are only a few studies focusing on the effect of medications on hospitalisation and mortality in patients with respiratory failure due to diseases other than COPD.^14^ Furthermore, newly published data from randomised studies show that LTMV in selected patients with COPD and respiratory failure can increase survival and reduce risk for hospitalisation,^15^ but data from ‘real-world’ patient cohorts are scarce. Morbidity and mortality are very high in patients with chronic respiratory failure.^16–18^ However, it is insufficiently explored how socioeconomic factors such as level of education, living conditions, marital status and national origin may impact treatment decisions, adherence to treatment and overall prognosis in this large patient group. Patients with obesity hypoventilation syndrome (OHS) have an increased usage of healthcare resources compared with controls^19^ and initiation of LTMV on small OHS cohorts has been associated with reduced healthcare utilisation.^20^ However, larger population-based studies on healthcare utilisation of patients with OHS on LTMV are needed.

In OSA, several pertinent questions are still unresolved. Observational studies have shown that treatment with CPAP is associated with increased survival.^2 21^ Randomised trials were not able to consistently show any secondary prevention effects of CPAP on cardiovascular events.^22–24^ Furthermore, it is yet unknown how day-time sleepiness in OSA may affect the risk of incident cardiovascular disease. A higher incidence of hypertension has been observed in sleepy patients with OSA compared with patients without day-time sleepiness.^25^ One well-cited study on a small sample size (n=441) of patients with the COPD-OSA overlap syndrome showed increased mortality and risk for hospitalisation for patients with COPD with untreated OSA compared with COPD alone.^26^ These results need to be verified in larger clinical-based studies.

The European Sleep Apnoea database (ESADA) study reported an increased cancer prevalence in women with OSA but not in men, but the reported cancer prevalence was low and no subanalysis for cancer subtypes was performed.^27^ Therefore, adequately powered large-scale studies of the possible association between OSA, gender and cancer are warranted.

Sweden offers extensive possibilities for population-based outcome studies. There are 107 national quality registries containing individualised data concerning specific diseases or medical interventions set up to monitor and to improve the quality of healthcare. The Swedish National Board of Health and Welfare (Socialstyrelsen) manages a series of registries relating to healthcare and social services. The Swedish system of personal identity numbers (PIN)^28^ allows identification of individuals in any national registry and cross-linkage between registries for longitudinal follow-up.

DISCOVERY is an acronym for Course of DISease in patients reported to the Swedish CPAP Oxygen and VEntilator RegistrY. In patients with chronic respiratory failure on LTOT and LTMV and with sleep apnoea on CPAP, important questions regarding comorbidity burden, hospitalisation rate, mortality and treatment outcomes are still unexplored to a large extent. The DISCOVERY cohort will provide unique opportunities by its size and comprehensiveness to fill this clinically relevant gap of knowledge.

### Cohort description

The DISCOVERY cohort is a nation-wide population-based cohort, including patients in the Swedevox National Quality Registry for respiratory failure, with respiratory failure starting LTOT (n=25 797), LTMV (both invasive and non-invasive ventilation, n=8111), patients with OSA starting CPAP (n=65 803) and a population-based control cohort (n=1 45 224).

#### National quality registry for respiratory failure (Swedevox)

Patients with LTOT and LTMV have been included prospectively in the Swedevox registry since 1 January 1987 and 1 January 1996, respectively, with 100% geographical coverage and a population-based coverage of approximately 85%–90%,^29–31^ even though temporary geographical gaps occasionally exist. The number of centres across Sweden reporting to the LTOT arm is 48 and the number reporting to the LTMV arm is 40. An increasing number of centres have prospectively included patients with OSA on CPAP therapy in the registry’s CPAP arm prospectively since inclusion started in 1 July 2010. The geographical coverage is estimated at 80% and the number of reporting centres across Sweden is 37 in 2018.^32^ The primary and secondary diagnosis causing treatment with LTOT and/or LTMV are reported as presented in tables 1 and 2. A complete variable list for the Swedevox registry is presented in table 3. Patients in the registry have scheduled follow-up visits after 1 year and those with LTMV also after 3 years. Follow-up frequencies are presented in table 4.

**Table 1:** Distribution of diagnosis and clusters of diagnosis in the LTOT arm of the DISCOVERY cohort (n=25 797)

Diagnosis n=	N (%)	Cluster	N (%)
COPD	15 716 (60.9)	Airway disease	17.256 (66.9)
Emphysema due to α1-antitrypsin deficiency	348 (1.3)		
Other airway disease	1192 (4.6))		
Pulmonary fibrosis	3627 (14.1)	Parenchymal disease	4522 (17.5)
Sarcoidosis	215 (0.8)		
Other parenchymal disease	680 (2.6)		
Primary pulmonary hypertension	580 (2.2)	Pulmonary vascular disease	1280 (5.0)
Chronic pulmonary embolism	480 (1.9)		
Other pulmonary vascular disease	220 (0.8)		
Heart disease	508 (2.0)	Heart disease	508 (2.0)
Thoracic deformation	703 (2.7)	Thoracic deformation	703 (2.7)
Hypoventilation	420 (1.6)	Hypoventilation	420 (1.6)
Pulmonary or pleural tumour	426 (1.7)	Pulmonary or pleural tumour	86 (1.7)
Other diagnosis	464 (1.8)	Other diagnosis	464 (1.8)
Waiting for definitive diagnosis	356 (1.4)	Waiting for definitive diagnosis	356 (1.4)

COPD, chronic obstructive pulmonary disease; LTOT, long-term oxygen therapy.

**Table 2:** Distribution of diagnosis and clusters of diagnosis in the LTMV arm of the DISCOVERY cohort (n=8111)

Diagnosis	N (%)	Cluster	N (%)
OHS with hypoventilation due to obesity and/or OSA	2764 (34.1)	OHS	2764 (34.1)
COPD	1103 (13.6)	Pulmonary disease	1451 (17.9)
Other pulmonary disease	348 (4.3)		
Status postpolio	381 (4.7)	RTD	980 (12.1)
Idiopathic scoliosis	373 (4.6)		
TBC sequelae	226 (2.8)		
ALS	1058 (13.0)	ALS	1058 (13.0)
Dystrophia myotonica	191 (2.4)	Neuromuscular disease	899 (11.1)
Duchenne’s/ Becker’s muscular dystrophia	175 (2.1)		
SMA	67 (0.8)		
Other neuropathies/ myopathies	466 (5.7)		
Spinal cord injury	179 (2.2)	Other diagnosis	848 (10.5)
Central hypoventilation with otherwise normal neurology	78 (0.9)		
Central hypoventilation secondary to brain injury/brain disease	163 (2.0)		
Other diagnosis	428 (5.3)		
Waiting for definitive diagnosis	111 (1.4)	Waiting for definitive diagnosis	111 (1.4)

ALS, amyotrophic lateral sclerosis; LTMV, long-term mechanical ventilation; OHS, obesity-hypoventilation syndrome; OSA, obstructive sleep apnoea; RTD, restrictive thoracic diseases; SMA, spinal muscular atrophy.

**Table 3:** Baseline variables and the variables at the 1-year and 3-year follow-up for the patients reported to Swedevox LTOT, LTMV and CPAP arms

Variables	LTOT Year 0	LTOT year 1	LTMV Year 0	LTMV year 1	LTMV year 3	CPAP Year 0	CPAP year 1
Treatment start date	+		+			+	
Date sleep study						+	
Date referral						+	
Gender	+		+			+	
Age	+		+			+	
PaO_2_ at air breathing (kPa)	+		+	+	+		
PaCO_2_ at air breathing (kPa)	+		+	+	+		
Saturation air (%)	+						
BE			+	+	+		
PaO_2_ at oxygen breathing (kPa)	+						
PaCO_2_ at oxygen breathing (kPa)	+						
Saturation (oxygen)	+						
Height (cm)	+		+			+	
Weight (m)	+		+	+	+	+	+
FEV_1_ (l)	+		+	+	+		
VC Supine			+	+	+		
AHI (events/hour)						+	
ODI (events/hour)						+	
ESS			+	+	+	+	+
Hypertension (Y/N)						+	
Main diagnosis	+		+				
Secondary diagnosis(is)	+						
Stable hypoxia at initiation?	+						
Smoking history	+						
WHO PS	+						
CAT score	+						
Liquid O_2_	+	+					
Type of oxygen equipment	+	+					
O_2_ dose (l/min)	+						
Treatment, hours/24 hours	+						
Days hospitalised			+				
Concomitant oxygen therapy			+	+	+		
Acute/elective initiation	+		+				
Treatment interphase (NIV/tracheostomy)			+	+	+		
Help needed with LTMV-interphase			+	+	+		
Date and reason of therapy termination		+		+	+		+
CPAP usage time (h/night)							+

AHI, Apnoea-Hypopnoea Index; PaO_2_ air, arterial blood gas tension of oxygen on air; BE, base excess; CAT, COPD assessment test; COPD, chronic obstructive pulmonary disease; CPAP, continuous positive airway pressure; ESS, Epworth Sleepiness Scale; FEV_1_, forced expiratory volume in 1 s; LTMV, long-term mechanical ventilation; LTOT, long-term oxygen therapy; ODI, oxygen desaturation index; PaO_2_ oxygen, arterial blood gas tension of oxygen on oxygen; PaCO_2_, partial pressure of arterial carbon dioxide; PS, performance status; VC, vital capacity.;

**Table 4:** Follow-up frequencies within the Swedevox registry for patients in the DISCOVERY cohort

	LTOT n (%)	LTMV n (%)	CPAP n (%)
Baseline	25 797 (100%)	8111 (100%)	65 803 (100%)
1-year follow-up	16 015 (62%)	4624 (57%)	25 854 (39%)
3-year follow-up	N/A	3088 (38%)	N/A

CPAP, continuous positive airway pressure; LTMV, long-term mechanical ventilation; LTOT, long-term oxygen therapy.

#### The control group

A population-based control group was identified from Statistics Sweden’s Living Conditions Surveys (ULF/SILC), which consists of a random selection of approximately 6000 people between the ages of 16 and 74 years in Sweden, made every year since 1987. The control cohort is from the same time span as the study population. Data from the ULF/SILC regarding age, gender, length, weight, smoking, housing type, living area, civil status and year of study are collected for the study.^33^

#### Compilation of the DISCOVERY data set

Within the Swedevox registry, there were patients reported to more than one registry arm and also patients that had initiated a specific treatment more than once (493 patients on LTOT, 141 on LTMV and 456 on CPAP). In the control group, 45 246 observations were from persons reported to ULF/SLIC on more than one occasion. The last registration in each arm was used in the subsequent analysis. In the control group, 1722 persons also reported to one of the arms in the Swedevox registry were excluded. The final DISCOVERY cohort consists of 25 804 unique patients with LTOT, 8111 with LTMV, 65 809 with OSA on treatment with CPAP and 145 224 persons in the control group. Characteristics of the different arms in the DISCOVERY cohort are presented in table 5.

**Table 5:** Characteristics of the DISCOVERY cohort

	LTOT n=25 797	LTMV n=8111	CPAP n=65 803	Control group n=145 224
Gender (male %)	11 846 (45.9)	4165 (51.4)	46 375 (70.5)	70 129 (48.3)
Age (years±SD)	73.3±9.8	60.6±16.0	57.2±12.5	49.9±20.4
BMI (kg/m^2^±SD)	24.6±6.5	32.9±10.8	31.9±6.2	24.9±4.0

**Figure 1:**
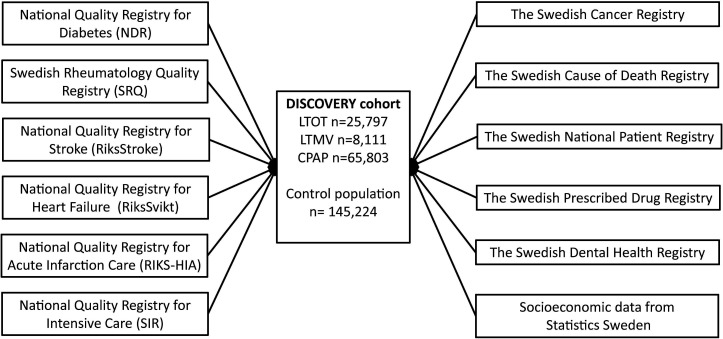
Sources of data for the DISCOVERY cohort. CPAP, continuous positive airway pressure; LTMV, long-term mechanical ventilation; LTOT, long-term oxygen therapy.

BMI, body mass index; CPAP, continuous positive airway pressure; LTMV, long-term mechanical ventilation; LTOT, long-term oxygen therapy.;

The Swedevox registry and the control cohorts were merged on individual level with governmental registries with almost complete coverage: the Swedish Cancer Registry, the Swedish Cause of Death Registry, the Swedish Drug registry, the Swedish National Patient Registry (NPR) and the Swedish Dental Health Registry. In addition, data were merged with the national quality registries for diabetes (NDR), for rheumatic diseases (Swedish Rheumatology Quality Registry (SRQ)), for stroke (RiksStroke), for heart failure (HF) (RiksSvikt), for acute heart infarction care (SwedeHeart) and for intensive care (SIR). Finally, socioeconomic data from Statistics Sweden (SCB) were added (figure 1).

**Figure 2:**
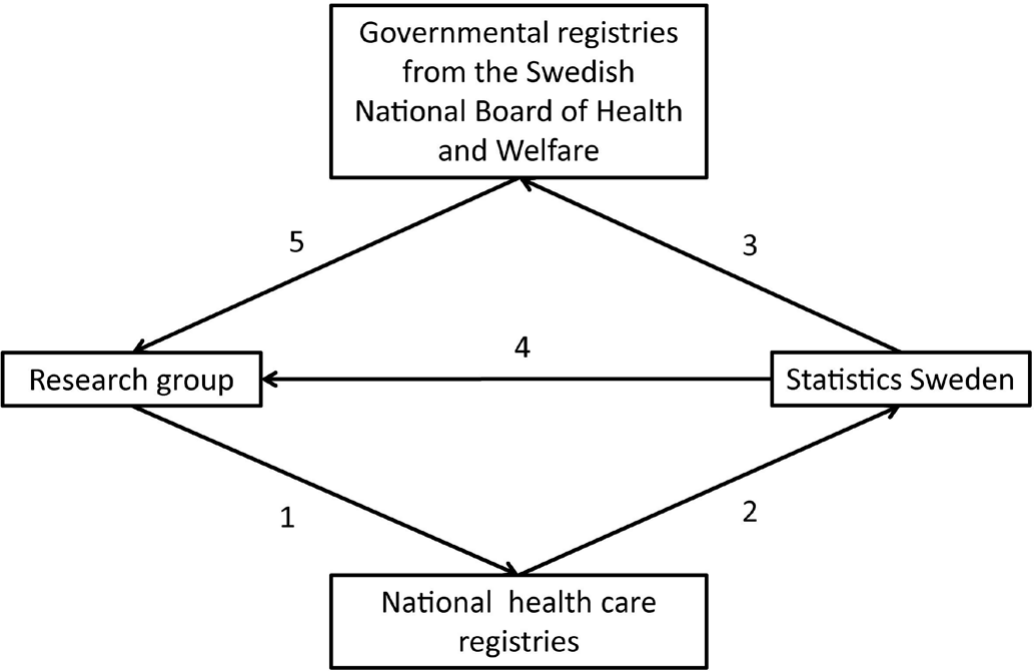
Flow chart for compilation of the DISCOVERY data set. The research group applied for extraction of data from the National healthcare registries (1). Data of the Swedevox population and from other national healthcare registries were sent to Statistics Sweden and a key file with personal identity numbers and unique serial numbers based on the Swedevox population was created (2). The key file and anonymised data from the other National Quality Registries were delivered to the Swedish National Board of Health and Welfare (3). Anonymised and serial number-labelled Swedevox and demographic data files and a data file of the control group file are delivered to the research group by Statistics Sweden (4). Anonymised and serial number-labelled data from the governmental and the other national quality registries were delivered to the research group by the Swedish National Board of Health and Welfare (5).

The national healthcare registries approved consignment of data. The stepwise process of cross-linkage is described in figure 2. All patients in the registries were labelled with unique serial numbers enabling merging of the different registries. The key file with personal identity numbers and serial numbers will be saved at the Swedish National Board of Health and Welfare for at least 3 years and thereby enable future corrections of errors, extraction of follow-up data and addition of other registries to the DISCOVERY cohort.

### Other national quality registries

#### National quality registry for diabetes

The Swedish National Diabetes Registry was launched in 1996 by the Swedish Society for Diabetology for the purpose of promoting evidence-based development of diabetes care. The registry includes risk factors, diabetic complications and medications. More than 90% of all adult patients with drug-treated diabetes type 1 and 2 in Sweden are registered.^34–36^

#### Swedish Rheumatology Quality Registry

The SRQ was started in 1995 by the Swedish Rheumatology Society to improve the healthcare and treatment of patients with rheumatoid arthritis (RA), but over time it has been expanded to cover several other rheumatic diseases including ankylosing spondylitis and psoriatic arthritis, myositis, systemic lupus erythematosus and additional conditions.^37^ This registry contains data on pharmaceuticals, diagnoses, inpatient care, specialised outpatient care, risk factors and follow-up data.^38^

The coverage for patients with RA on biologic treatment is 95% and patients with ankylosing spondylitis, psoriatic arthritis and other spondyloarthropathies on biologic treatment is 86%.^39^ Coverage for all patients with RA is estimated at 83.9% nationwide in 2017.^40^

#### National quality registry for stroke (RiksStroke)

RiksStroke was established in 1994 and has complete geographical coverage^41^ and a completeness of 89%. It registers patients with acute stroke (acute ischaemic stroke, intracerebral haemorrhages and since 2010 also patients with transitory ischaemic attacks (TIA)).^42^ By 31 December 2018, 531 156 patients have been included into the registry.^43^

#### National quality registry for heart failure (RiksSvikt)

Patients with HF, both in primary care and in hospital-based care, are reported to the RiksSvikt registry. The registry was founded in 2000 and implemented throughout Sweden in 2003.^44^ The registry contains information on underlying diseases, diagnoses, treatment, quality of life, functional capacity, laboratory values, blood pressure, heart rate and planned follow-up.^45^

By 2014, 63 519 unique patients have been included into the registry and there are now approximately 10 000 registrations added every year. Coverage of prevalent HF in the inpatient setting is 54% but much lower in primary care.^46^

#### National quality registry for enhancement and development of evidence-based care in heart disease (Swedeheart)

Since 1995, patients with acute coronary disease are reported to the national quality registry for acute infarction care (RIKS-HIA), a registry which since 2008 is a part of the Swedeheart registry. All coronary care units in Sweden register patients suffering from myocardial infarction and patients undergoing coronary angiography/angioplasty or heart surgery for any indication. Approximately 80 000 patients are reported annually.^47–49^

#### National quality registry for intensive care (SIR)

Since 2005, Swedish intensive care units have reported patient data to Swedish Intensive Care Registry. The geographical coverage is 92%. This registry contains data on diagnoses, interventions, inpatient care, patient-reported outcome measures (PROM)

### Governmental registries administrated by the Swedish National Board of Health and Welfare

#### The Swedish Cancer Registry

The Swedish Cancer Registry was founded in 1958 and has an estimated completeness of 96% for all diagnosed cancers in Sweden with approximately 60 000 new malignant cancer cases are reported annually.^51 52^ It is mandatory for all healthcare providers to report all new cancer cases to the registries. The basis for diagnosis can be clinical examination, histology/cytology, surgery, autopsy or other examinations such as CT/MRI or laboratory investigations. Information about anatomic site and histological type and stage of the cancer, and date of diagnosis is recorded. The type of malignancy has been coded according to the Swedish versions of the International Classification of Disease system.^53^

#### The Swedish Cause of Death Registry

Since 1952 all deaths of persons in Sweden are registered in the Swedish Cause of Death Registry.^54^ Up to 96% of persons included have a specific underlying cause of death recorded.^55^

#### The Swedish Prescribed Drug Registry

The Swedish Prescribed Drug Registry contains data on all dispensed prescriptions in outpatient care in Sweden since 1 July 2005.^56 57^ All drugs are classified according to the Anatomical Therapeutic Chemical classification system.^58^

#### The Swedish National Patient Registry

Data about comorbidities, hospitalisations, operations and other procedures were derived from the National Patient Registry (NPR) for inpatient and outpatient care.^59^ The inpatient registry, also called Hospital Discharge Registry (NPR) was established in 1964 and has complete geographical coverage since 1987. The NPR covers nearly all somatic and psychiatric hospital admissions and as of 2001 is virtually 100% complete for data from public hospital-based outpatient care givers. The overall completeness for outpatient care is 80% due to missing data from private caregivers. Primary care is not covered in the NPR.^60^

#### The Swedish Dental Health Registry

The Swedish Dental Health Registry was initiated in 1 July 2008 and contains individual data on dental healthcare to the whole adult population of Sweden.^61^ The registry contains data on diagnosis, number of remaining and intact teeth, examination and treatment forms.^62^

#### Demographic data from Statistics Sweden

The Swedish Longitudinal Integrated Database for Health Insurance and Labour Market Studies (LISA) contains annual data on county and place of residence, civil status, migration, country of birth, foreign background and nationality, length of education, occupation and employment variables, incomes and allowances, sick leave and incomes. LISA covers the adult Swedish population aged ≥16 years registered on 31 December each year since 1990. The completeness is estimated at 95%.^63^ Data were also retrieved from Statistics Sweden on housing conditions.

Patients in the DISCOVERY cohort were continuously followed-up when being registered in other national quality registries or in the mandatory governmental registries due to death, when receiving a cancer diagnosis, when being prescribed medications, when being hospitalised, when receiving outpatient care at a public hospital and when receiving publicly financed dental care. There is generally follow-up data available until 12 March 2018. For the DISCOVERY cohort, there is longitudinal data available up to 31 December 2017 in the national cancer and the national cause of death registries and up to 31 December 2018 in the National Patient, National Prescribed Drugs and the National Dental Health registries. The frequency the study population appeared in the other registries is presented in table 6. No patients except those who emigrate are lost to follow-up due to the cross-linking with mandatory governmental registries.

**Table 6:** Cross-tabulation of the Swedevox registry population and control population in relation to national quality registries and mandatory governmental registries

	Time period	LTOT n=25 797	LTMV n=8111	CPAP n=65 803	Control group n=145 224	Main variables
**Swedish national quality registries**						
National quality registry for diabetes n=33 319 unique patients	2 Jan 1997–4 Apr 2019	3002	2160	15 101	13 056	Anthropometrics, biochemical analysis including HbA1c, physical activity, smoking and snuff habits, blood pressure, complications to diabetes (retinopathy, albuminuria, foot complications).
Swedish Rheumatology Quality Registry n=1584 unique patients	20 Aug 2009–20 Jan 2020	99	46	630	809	Pharmaceuticals, diagnoses, inpatient care, specialised outpatient care, risk factors and follow-up data.
National quality registry for stroke n=9149 unique patients	1 Jan 2001–31 Dec 2017	1057	412	2152	5528	Pharmaceuticals, risk factors (atrial fibrillation, diabetes, smoking habits), length of hospitalisation, details about thrombolysis and medical investigation.
National quality registry for heart failure n=4712 unique patients	9 Mar 2002–17 May 2019	1119	457	1460	1676	Anthropometrics, pharmaceuticals, smoking habits, aetiology of heart failure, cardiovascular comorbidities, NYHA function class, ultrasonographic findings, cardiac implantable electronic device therapy.
National quality registry for acute infarction n=23 164 unique patients	23 Jan 1991–17 Dec 2018	4394	1263	6470	11 037	Anthropometrics, pharmaceuticals, smoking and snuff habits, medical history, biochemical analysis, reperfusion therapy, electrical devices, stress test, echocardiography, CPAP therapy, length of hospitalisation.
National quality registry for intensive care n=14 700 unique patients	1 Jan 2008–19 Mar 2019	3604	2436	3776	4884	Level of hospital (primary, secondary or tertiary), duration of hospitalisation, time of discharge, operation (Y/N, elective/acute), discharged alive (Y/N), diagnosis, decision to forego life-sustaining therapy (Y/N), mechanical ventilation (Y/N), cardiovascular, hepatic, gastroenterological, neurological, renal, respiratory, haematological, metabolic, traumatic or other reason for ICU care.
**Swedish governmental registries**						
National Cancer Registry n=38 291 cancer diagnosis	5 Jan 1987–31 Dec 2017	4984	1355	6904	25 048	Date and ICD number of cancer diagnosis.
National Cause of Death Registry n=60 763 deaths	1 Jan 1996–31 Dec 2018	23 573	4970	2786	29 434	Date of death, primary cause of death, contributing cause(s) of death.
National Patient Registry						
Inpatient registry n=9 52 348 care events	1 Jan 1987–31 Dec 2017	288 024	103 641	143 198	417 485	Date of hospitalisation and discharge, hospital code, main diagnosis, secondary diagnosis(is), surgical diagnosis(es).
Outpatient registry n=3 736 276 visits	1 Jan 2001–31 Dec 2017	388 501	264 873	1 100 001	1 982 901	Date of visit, hospital code, main diagnosis, secondary diagnosis(es), surgical diagnosis.
National Prescribed Drugs Registry n=14 519 303 drug prescriptions	14 March 2005–31 Dec 2018	1 727 676	911 168	4 650 203	7 230 256	Prescription date, expedition date, ATC-code, information about all dispensed prescriptions, cost.
National Dental Health Registry n=2 869 575 visits	1 Jul 2008–30 Dec 2018	74 468	51 812	982 615	1 760 684	Date of visit, number of intact and remaining teeth.

Time period is 5 years prior to inclusion into the Swedevox registry until end of study period.

ATC-code, Anatomical Therapeutic Chemical code; CPAP, continuous positive airway pressure; HbA1c, glycated haemoglobin; ICD, International Classification of Disease; ICU, intensive care unit; LTMV, long-term mechanical ventilation; LTOT, long-term oxygen therapy; NYHA, New York Heart Association.

### Findings to date

Several studies from the Swedevox registry have previously been published and a complete publication list can be found in the Swedevox annual report 2018.^32^ In patients with COPD and respiratory insufficiency, pharmacological treatment for cardiovascular diseases^64^ and high doses of benzodiazepines and/or opioids^65^ associates with increased risks for hospitalisation and mortality in contrast to patients with COPD without respiratory insufficiency.^66 67^ In interstitial lung disease, no association has been found between benzodiazepines and/or opioids and risk for mortality and hospitalisation.^14^ Previous studies from the LTOT arm have shown that despite improved oxygen and pharmacological treatment with bronchodilators in patients with COPD, the age-adjusted survival in patients with LTOT has not improved.^12^ Characteristics of patients receiving LTOT has changed markedly over time. Since the first LTOT studies^68 69^ and since 1987, when the Swedevox registry was launched, mean age at initiation of LTOT has increased by approximately 10 years; a majority of patients receiving LTOT are women and the burden of comorbidities has increased.^12 32 70^

**Figure 3:**
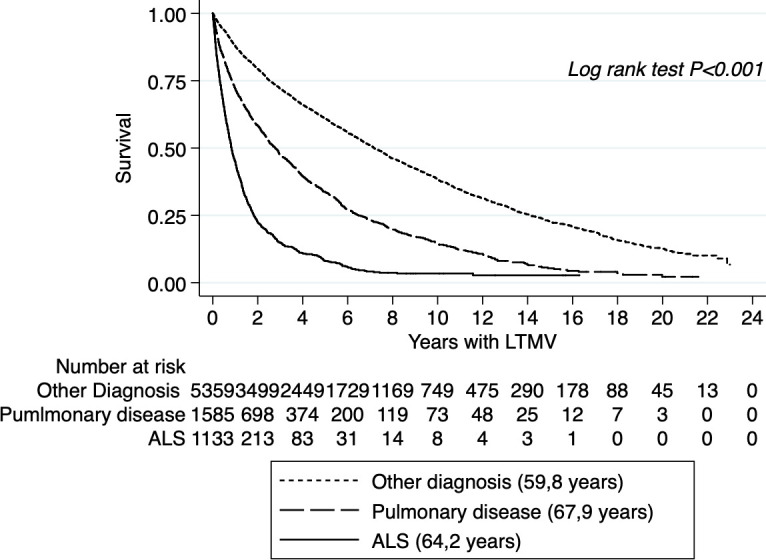
Kaplan-Meier estimate showing heterogenicity of survival in patients with LTMV reported to the Swedevox registry 1996–2018. Mean age for each group is stated in the legend. ALS, amyotrophic lateral sclerosis; LTMV, long-term mechanical ventilation.

The LTMV group is very inhomogeneous with differences in survival and mean age between groups as a consequence (figure 3). A previous study on Swedevox data has shown that women with OHS are older and more obese, and they have a more advanced OHS and more often initiate LTMV under non-elective conditions than men.^17^ Female gender and presence of hypertension were associated with non-adherence to CPAP therapy, whereas higher age, more severe OSA, BMI up to 35 and use of a humidifier were associated with adherence.^18^ The mortality among those with non-adherence was nearly doubled. A recently submitted study from the DISCOVERY cohort showed that civil status, educational level, household income and foreign background are strong predictors for adherence to CPAP therapy in patients with OSA.^71^

Thus, the DISCOVERY database will be used to study factors associated with incidence, progression, worse quality of life, hospitalisation, comorbidity and mortality in patients with chronic respiratory failure with LTOT, LTMV and OSA on CPAP treatment. The detailed planning is ongoing.

### Strengths and limitations

This nationwide population-based cohort is unique internationally due to its size, its high nationwide coverage and its high degree of data completeness. A recent validation study support that Swedevox data are valid, of high quality and suitable for use in research.^72^ No patients are lost to follow-up due to cross-linkage with mandatory national registries. Another important strength is the large population-based control group collected during the same time period as the Swedevox patients. The Swedish system with personal identity numbers enables linking of individuals in any national registry which provides the opportunity to create a unique database allowing analysis of longitudinal data from different data sources. A number of limitations need to be mentioned. To assure a high level of completeness in the Swedevox registry, the number of requested variables to be reported into the Swedevox registry has been deliberately limited, and the data thus become less detailed. This is counterbalanced, however, at least in part, by the size of the cohort. The assessment of comorbidities may be less detailed as only hospital-based diagnosis are captured in the final DISCOVERY database. However, data from drug prescription and drug utility pattern on each individual patient allow for a precise adjustment of concurrent drug treatment. It might be argued that this procedure may have the advantage of more precise control of comorbidities as we can adjust for only the more severe, drug-treated conditions. Another limitation is that the national quality registries are not mandatory with varying coverage as a consequence. Difficulties adjusting for confounders is a limitation in all registry-based studies.

## Collaboration

Requests for specific research projects and collaborative work are encouraged and can be addressed to the corresponding author.

### Patient and public involvement

A representative from the patient organisation ‘the Swedish Heart and Lung Association’ is a member of the Swedevox Registry’s steering committee and has been involved in study design discussions. Results from future studies from the DISCOVERY cohort will be submitted for publication in peer-reviewed journals and presented at relevant conferences.
